# A 5-year limb salvage for long-term limb salvage in chronic limb-threatening ischemia without options for revascularization, utilizing a hybrid deep venous arterialization technique: A case report

**DOI:** 10.1177/00368504251352033

**Published:** 2025-06-25

**Authors:** Lucas Ferrer, Lynsey Malin, Panagiotis Koutakis, Dimitrios Miserlis

**Affiliations:** 1 Vascular and Endovascular Surgery, 377659 University of Texas at Austin Dell Medical School, Austin, TX, USA; 2 Department of Public Health, College of Health, 6491 University of West Florida, Pensacola, FL, USA

**Keywords:** Deep venous arterialization, peripheral arterial disease, limb ischemia, limb salvage, small artery disease, desert foot

## Abstract

Chronic limb-threatening ischemia represents the most severe form of peripheral arterial disease, with a 40% amputation rate and 20% mortality at 6 months without revascularization. Deep venous arterialization is an alternative for patients with advanced peripheral arterial disease who have no conventional revascularization surgery option based on their anatomy—no distal artery target to redirect arterial blood. Hybrid deep venous arterialization, which combines open surgical bypass with venous endovascular arterialization and valvulotomy, is a technique under investigation for limb salvage with limited current long-term data. We present a successful limb salvage case report of an early 60s male with no conventional revascularization options who presented with a wound in his foot and underwent a successful hybrid deep venous arterialization. The hybrid deep venous arterialization technique has shown promising preliminary results for long-term limb salvage at 5 years after surgery. This case report establishes a tangible example of the potential role of hybrid deep venous arterialization in the management of patients with no-option chronic limb-threatening ischemia. After a 5-year follow-up, the reported patient has no ischemic symptoms, and he is able to ambulate without support.

## Introduction

Peripheral arterial disease (PAD) poses a significant health challenge globally, affecting over 200 million people worldwide.^1^ The restricted blood flow to the extremities leads to tissue architectural deterioration, metabolic dysfunction, and symptoms such as pain, numbness, and impaired mobility.^2,3^ Conventional revascularization techniques involve: open bypass surgery, endovascular revascularization, and hybrid revascularization, redirecting blood to a distal patent artery utilizing a combination of open and endovascular surgery methods. Critical limb-threatening ischemia (CLTI) is the most severe form of PAD, characterized by ischemic rest pain, ulcers, or gangrene.^4^ Ferraresi et al.^5^ demonstrated the role of small artery disease (SAD) as the leading factor in CLTI and described it as a failure to distribute blood flow to the feet, leaving the foot with only collateral vessels (“desert foot”) and a lack of available arterial outflow targets for treatment. Without revascularization, the amputation rate is as high as 40% and mortality as high as 20% at 6 months after onset of CLTI symptoms.^6^

In 1977, Sheil performed arteriovenous anastomosis successfully, with the resolution of pain in five of six patients with no-option CLTI except an amputation.^7^ Deep venous arterialization (DVA) creates a connection between a proximal arterial inflow and a distal deep venous target at the ankle with concurrence disruption of the vein valves. The hybrid DVA technique combines open surgical bypass and an endovascular approach of valvulotomy of distal collateral veins either immediately or in the weeks following.^7^ We present a successful case of limb salvage after a hybrid DVA for a no-option CLTI. The patient has provided written informed consent for the publication of this case report and any accompanying images.

## Case report

**Figure 1.**
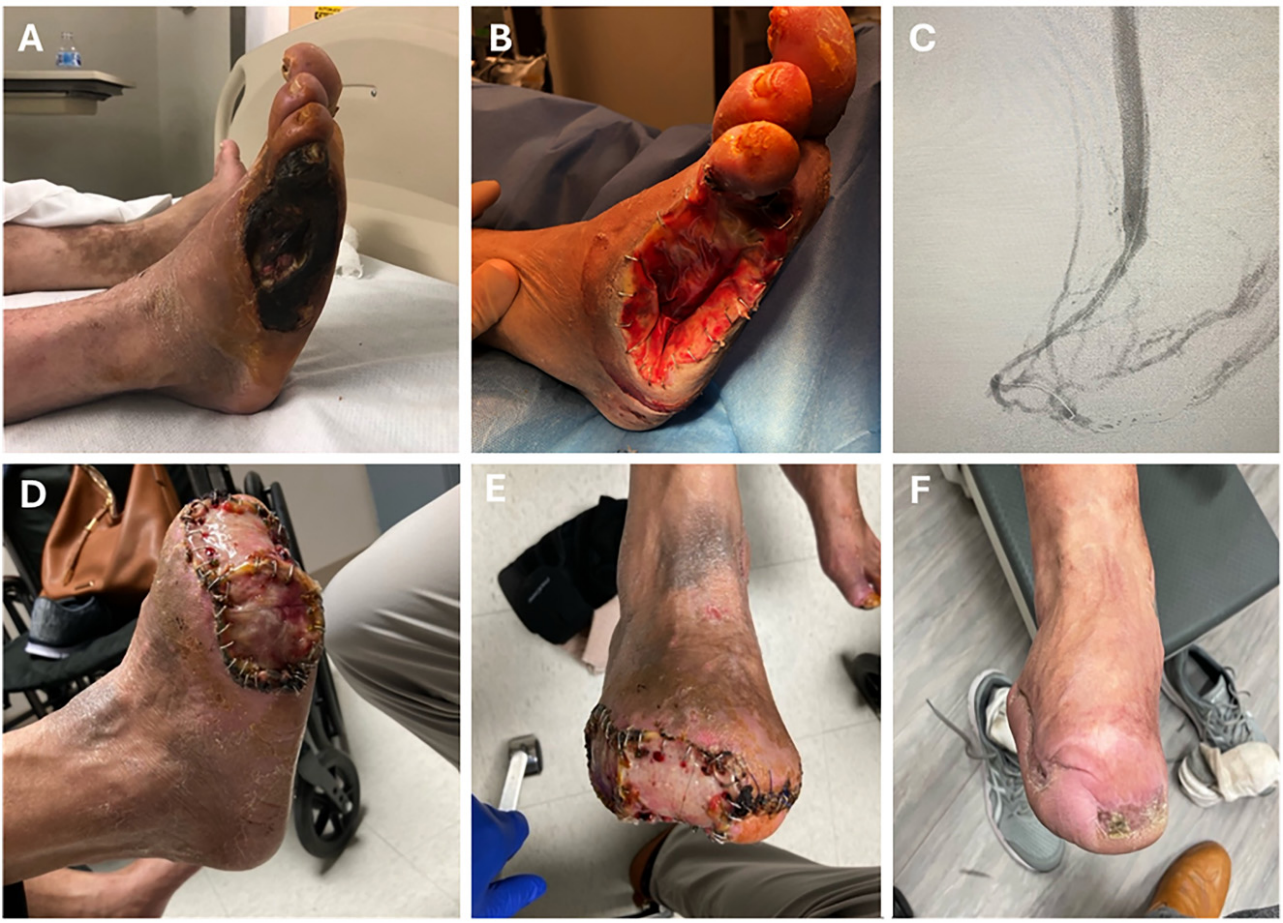
Imaging of this case (a) initial wound with gangrenous toes; (b) initial debridement and amputation of fourth and fifth toes; (c) angiogram of venous arterialization; (d) foot reconstruction with dermal matrix; and (e, f) heal reconstructed wound 5 years after the venous arterialization procedure (right leg).

This case involves a patient in the early 60s-year-old, male, with CLTI and a history of left fifth toe and need for right fourth and fifth toe amputations with multiple comorbidities, including uncontrolled diabetes mellitus type 2, coronary artery disease, controlled hypertension, hyperlipidemia, end-stage renal disease on hemodialysis, and former smoking history. The patient's medical history included apixaban (5 mg twice a day), atorvastatin (40 mg daily), aspirin (81 mg) daily, insulin (0.5 units per kg), tirzepatide (15 mg weekly), losartan (50 mg daily), metoprolol (100 mg daily), and furosemide (60 mg daily). His non-healing ulcers on his right fourth and fifth toes were Rutherford class 6 with dry gangrene and WIfI classification of 4^8^ (Figure 1(a)). The toes had discoloration and minimal purulent drainage, without systemic signs of fever or leukocytosis. The patient had palpable femoral pulses and no palpable pulses at the foot level. The patient was ambulating with a walker due to lower extremity pain with exertion and climbing stairs. The patient had an amputation for source control of his right fourth and fifth toes (Figure 1(b)).

An angiogram was performed which demonstrated no distal target for revascularization (no pedal/tarsal target and no long segment greater saphenous vein available), with scarce presence of foot arterial arch vessel, characteristic of a desert foot due to small artery disease (SAD). It was determined that conventional revascularization was not an option and the patient instead we selected venous arterialization. At the time of the intervention full endovascular DVA as a technique was not yet established in the United States and based on our surgical experience we selected a hybrid DVA. The procedure was performed under general anesthesia, with prophylactic antibiotics and perioperative heparin administration. A bypass was performed from the popliteal artery to the great saphenous vein at the malleolus level using an Artegraft graft (Artegraft, North Brunswick, New Jersey). Manual valve lysis of the medial marginal vein valves was done with coronary dilators (mechanical destruction of the valves). All collaterals above the ankle underwent side branch ligation. After the open portion of the surgery, the distal vein valves were broken endovascularly to redirect the flow at the venule-capillary level (Figure 1(c)).

In a secondary procedure three days later, endovascular venous arch valve lysis and focalization of flow into the forefoot with coiling of stealing venous collaterals was performed. Blood flow was focused on the wound by embolizing proximal foot vein collaterals to create forward pressure. A follow-up ultrasound demonstrated severe stenosis at the medial marginal vein and bypass of the DVA anastomosis. He then underwent a serial angioplasty of the medial marginal vein, followed by a coil of the major tributary vein that was directing flow away from the forefoot.

The patient was placed on clopidogrel post-operatively (75 mg daily). During the same hospitalization, the patient underwent a modified transmetatarsal amputation of his right first and second toes with partial third toe amputation, as well as partial resection of the fifth metatarsal with excisional debridement to the bone with wound vacuum placement (Figure 1(d)). In our experience and literature, perfusion/TcPO2 increases at 6 weeks, and at that point healing usually progresses well, we were forced to proceed with early amputations for source control of infection. He achieved complete healing 8 weeks afterwards; followed by right lower extremity wound debridement with split-thickness skin graft and Integra placement (Integra LifeSciences, Plainsboro, New Jersey). Six weeks later the wound vacuum was taken down revealing healthy tissue at the skin graft site. The clinical and laboratory follow-up demonstrated notable improvements in pain reduction, wound healing, and blood perfusion. Five years after his procedure the patient is free of ulcers with a TcPO_2_ of 46 (Figure 1(e) and (f)). The patient reports an overall improved quality of life and increased capacity to perform activities of daily life. The patient has provided a written informed consent form for treatment, and he was admitted to Texas at the affiliated hospital facility in June 2020. The reporting of this case adheres to the CARE guidelines.^9^

## Discussion

DVA is an option when symptoms are not improved despite excellent wound care, medications, and attempts to revascularize or if revascularization is not an option given the lack of a distal target and a desert foot.^7^ The exact mechanism of action of this technique is unknown but appears to have a component of neoangiogenesis. The favorable results observed in this case underscore the potential of DVA as a viable therapeutic option for CLTI patients with Rutherford class 5 or 6 disease without conventional revascularization options.

The hybrid DVA technique combines the benefits of open and percutaneous approaches for DVA. It consists of the creation of a conventional proximal anastomosis between the inflow artery and vein in an open fashion, followed by endovascular creation of distal venous outflow with valvotomy or embolization of the distal collateral venous branches.^7^ Ferraresi et al.^10^ demonstrated good short-term patency outcomes in a case series of 35 CLTI patients undergoing hybrid DVA, with an 86.1% primary patency rate and 91.7% secondary patency rate at 1-month follow-up. Follow-up at 1 year showed a dramatic decrease to 6.9% primary patency rate and 8.1% secondary patency.

Currently, the most prominent study for venous arterialization available is the Promise II Trial.^11^ The study focuses on patients with no-option CLTI, utilizing a proprietary catheter-based system for endovascular surgery (LimFlow SA) for DVA. Encompassing over 100 participants across the United States, the trial included individuals with CLTI rest pain, nonhealing wounds, diabetes, chronic kidney disease, and those on dialysis. This study achieved a 66% rate of amputation-free survival at six months, demonstrating the potential of endovascular DVA in a diverse patient population.^11^ However, longer-term data is not yet available.

Further research is warranted to establish the long-term efficacy and safety of this approach and technique optimization. The underlying mechanisms, ischemia-reperfusion, metabolism, tissue remodeling, and wound healing biology, as well as the technical minutia still require evaluation. Comparative studies with larger sample sizes and extended follow-up periods will provide a more comprehensive understanding of the benefits and limitations of venous arterialization, as there is a lack in the literature of long-term efficacy or extended follow-up beyond the 1-year follow-up reported.

## Conclusion

The hybrid DVA technique has shown promising preliminary results, especially in patients with Rutherford class 5 or 6 disease. The positive long-term outcomes at 5 years observed in this case report establish a tangible example of the potential role of hybrid DVA in the management of patients with no-option CLTI. However, long-term efficacy and safety, technique optimization, underlying biology mechanisms investigation, as well as comparative trials with larger sample sizes, are necessary to fully understand the benefits and limitations of venous arterialization.

## Learning points

For patients with an open wound in the foot, it is important to act fast in order to optimize limb salvage:

- For wound infection, proceed with debridement and necessary amputation, for example, toes, and transmetatarsal, early for source control in order to avoid below-knee or above-knee amputations.
- If the patient has PAD and no option for conventional revascularization there is still potentially an option for venous arterialization, currently offered by specialized institutions and it is important to refer early.
- The technique of hybrid revascularization is one method for DVA in patients with SAD and dessert foot, as reported in our case lead to a 5-year limb salvage, however, the technique is still under investigation.
